# Genotypic study of isolated resistance to isoniazid in the Mycobacterium tuberculosis complex in a Moroccan hospital

**DOI:** 10.1099/acmi.0.000928.v5

**Published:** 2025-08-08

**Authors:** Amine Amri, Elmostafa Benaissa, Yassine Benlahlou, Fatna Bsaibis, Adil Maleb, Mariama Chadli, Mostafa Elouenass

**Affiliations:** 1 Mohammed V Military Instruction Hospital, Rabat, Morocco; 2 Mohammed VI University Hospital, Oujda, Morocco

**Keywords:** diagnostic, *inhA*, *katG*, mutation, resistance, tuberculosis

## Abstract

**Introduction.** Despite the introduction 40 years ago of effective and low-cost treatment for tuberculosis (TB), morbidity and mortality from this disease remain substantial worldwide. According to the WHO, TB is once again the leading cause of death worldwide from a single infectious agent. In 2023, TB caused ~1.25 million deaths, surpassing COVID-19. In Morocco, the number of new TB cases rose from 30,897 in 2017 to 35,000 in 2019, highlighting a concerning upward trend that underscores the persistent challenge TB poses to the country’s public health system. The incidence of multidrug-resistant (MDR) or rifampicin (RIF)-resistant TB was estimated at 1.7 per 100,000 inhabitants. Isoniazid (INH) is a cornerstone of first-line TB treatment, and resistance to it, even in the absence of RIF resistance, is associated with delayed treatment response, higher rates of treatment failure or relapse and increased risk of progression to MDR-TB if not promptly identified and appropriately managed. Moreover, current diagnostic algorithms in many settings, including Morocco, may miss INH monoresistance due to their reliance on rapid molecular tests that primarily detect RIF resistance, further emphasizing the emerging threat of drug-resistant TB. Despite this, national data on INH monoresistance remain scarce. Given the increasing burden of TB and the critical importance of early detection of drug resistance, it is essential to better understand patterns of resistance beyond RIF. It is within this context that we conducted the present study, which aims to investigate INH resistance in TB cases (pulmonary or extrapulmonary, new or previously treated) over a period of 3 years.

**Materials and methods.** This is a retrospective study conducted at the Bacteriology Department of Mohammed V Military Instruction Hospital over a period of 3 years. Data were collected via the laboratory information system. Clinical samples underwent treatment using both conventional bacteriological methods and molecular techniques. The study of resistance to major anti-TB drugs was performed using the reverse hybridization technique, specifically the HAIN method (GenoType^®^ MTBDR plus by Hain Lifescience). Statistical analysis was performed using IBM SPSS Statistics 19 and Microsoft Excel 2019.

**Results.** The study involved 464 patients treated for pulmonary and extrapulmonary TB, including both new cases and those previously treated with positive cultures. The mean age of the patients was 42.2 years, with a range from 8 to 88 years. There was a predominance of males at 74%, with a sex ratio of 2.8.

Pulmonary sputum samples accounted for 84.8% of the cases, whereas extrapulmonary samples represented only 15.2%, and the positivity rates for direct examination and culture across all samples were 74% and 100%, respectively. INH resistance had a prevalence of 9% (43 out of 464). Genetic mutations observed indicated that 63% of the clinical isolates resistant to INH had mutations in the *katG* gene, while 37% had mutations in the *inhA* gene.

**Conclusion.** The increasing prevalence of *Mycobacterium tuberculosis* complex strains resistant to one or more first-line anti-TB drugs highlights the urgent need for targeted and ongoing epidemiological surveillance. In this study, we found that INH resistance affected 9% of TB cases over the 3-year period, underscoring a significant yet under-recognized threat to TB control efforts in Morocco. Molecular analysis revealed that the majority of resistant strains carried mutations in the *katG* gene, with a smaller proportion exhibiting mutations in the *inhA* promoter region. These findings emphasize the importance of incorporating molecular diagnostics capable of detecting INH resistance even in the absence of RIF resistance into routine TB surveillance programmes. Strengthening diagnostic capacity and updating treatment protocols accordingly will be essential to curb the spread of INH-resistant TB and prevent the emergence of MDR forms.

## Data Summary

All data associated with this work is reported within the article.

## Introduction

Tuberculosis (TB) is a transmissible disease ranked among the leading causes of ill health and is one of the foremost causes of death worldwide. Prior to the emergence of the COVID-19 pandemic, TB was the leading cause of death attributable to a single infectious agent, surpassing human immunodeficiency virus (HIV). According to the World Health Organization (WHO), TB is once again the leading cause of death worldwide from a single infectious agent. In 2023, TB caused ~1.25 million deaths, surpassing COVID-19 [1].

In Morocco, 35,000 new cases of TB were recorded in 2019 (the number of cases was 30,897 in 2017), while the incidence of multidrug-resistant (MDR) or rifampicin (RIF)-resistant TB was estimated at 1.7 per 100,000 inhabitants, with extrapulmonary TB accounting for 48% according to the National Tuberculosis Control Program.

In Morocco, according to the WHO estimates in 2022, 1.2% of TB cases (410 cases) and 2.7% of TB-related deaths (73 deaths) are HIV-positive. A national survey conducted in 2008 on the prevalence of HIV positivity among TB patients revealed a national rate of 1.7%, with regional rates reaching up to 8.2% in the Souss-Massa region. National data from HIV screening activities among TB patients confirm the results of the aforementioned HIV seroprevalence survey. Thus, the HIV positivity rate among TB patients with known HIV status ranged from 1.4% to 1.9% between 2016 and 2022. Among the 266 patients with TB/HIV co-infection reported in 2022, 58% had a known positive HIV status prior to the TB diagnosis, and 42% were found to be HIV-positive following routine HIV screening among TB patients [2]. According to the 2022 report from the WHO, ~8.5% of newly diagnosed TB cases worldwide exhibit resistance to isoniazid (INH). The emergence and spread of drug-resistant TB are now recognized as one of the most dangerous threats to the global fight against TB [3]. Patients with drug-resistant TB, including MDR strains resistant at least to RIF and INH, or extensively drug-resistant with the MDR phenotype plus resistance to at least one fluoroquinolone and one of the three second-line injectable drugs (kanamycin, capreomycin and amikacin), require long, toxic and expensive treatment with unsatisfactory outcomes [4,5]. The emergence of resistant strains justifies the determination of drug sensitivity in all cases of TB. For this, two types of methods are available: phenotypic methods, which rely on detecting the growth of *Mycobacterium tuberculosis* strains on culture media supplemented with antibiotics, and genotypic or molecular methods, which rely on detecting mutations in target genes known to be responsible for resistance to anti-TB drugs. The use of genotypic methods that detect mutations in the genes involved in drug resistance of *M. tuberculosis* is advantageous because the results are available within a few days, whereas with phenotypic methods for determining antibiotic sensitivity, results take anywhere from 5 to 21 days (proportion methods). Most genotypic methods rely on a PCR amplification of a gene region or the entire gene involved in resistance. Subsequently, the detection of mutations can be carried out using various methods [6]. At the national level, there are few studies focusing on INH monoresistance. Therefore, this study aims to investigate INH resistance in TB cases (pulmonary or extra-pulmonary, new cases or previously treated) over a period of 3 years.

## Methods

### Study design and setting

This is a retrospective study conducted in the bacteriology department of the Mohammed V Military Hospital, spanning a period of 3 years, from March 2019 to December 2023, involving 464 patients who were followed for pulmonary or extra-pulmonary TB, either new cases or previously treated, with positive cultures.

### Laboratory methods

The laboratory was equipped with the following:

For microscopy: a binocular microscope (Olympus CX31^®^ model), along with standard Ziehl–Neelsen staining supplies and reagents, including acid, alcohol, fuchsin, methylene blue, filters, glass slides and cotton.

For culture: solid media including Löwenstein–Jensen (LJ), LJ supplemented with 0.2% sodium pyruvate and LJ containing thiophene-2-carboxylic acid hydrazide. Reagents used included 4% sodium hydroxide, nitrates, sodium nitrate, Griess reagents A and B, hydrogen peroxide and Tween 80.

Culture preparation involves growing *M. tuberculosis* on solid or liquid media (e.g. LJ or MGIT). Spreading samples on slides followed by Ziehl–Neelsen staining is part of smear microscopy and serves as a follow-up test for post-culture confirmation. Molecular Methods for Identification and Drug Resistance Detection: the GenoType^®^ MTBDR plus test enables the identification of the *M. tuberculosis* complex and the detection of resistance to RIF and/or INH after DNA amplification. This test can be performed on mycobacterial isolates cultured on either solid or liquid media. It can also be applied directly to clinical pulmonary specimens, regardless of whether the smear microscopy is positive or negative. RIF resistance is identified by detecting the most significant mutations in the *rpoB* gene, which encodes the β-subunit of RNA polymerase. High-level INH resistance is detected through the analysis of mutations in the *katG* gene, which encodes the enzyme catalase-peroxidase. To assess low-level INH resistance, the promoter region of the *inhA* gene is analysed. This gene encodes the enzyme NADH-dependent enoyl-ACP reductas**e**.

The validation of the GenoType MTBDR plus test results follows a rigorous process. Before the test is used systematically in clinical settings, its performance is validated in the laboratory by comparing the obtained results with reference methods, such as bacterial culture and drug sensitivity tests. This validation ensures that the mutations detected by GenoType MTBDR plus correlate with drug resistance. Quality controls are regularly performed to ensure that the test is functioning correctly and that the results are reliable. This includes the use of validated reagent lots and ensuring that the analysis procedures comply with standardized protocols. The quality control was conducted using the reference strain *M. tuberculosis* NCTC 7416, H37Rv.

### Data analysis

Data collection was performed from the laboratory information system, and statistical analysis was performed using IBM SPSS Statistics 19 and Microsoft Excel 2019.

## Results

**Fig. 1.**
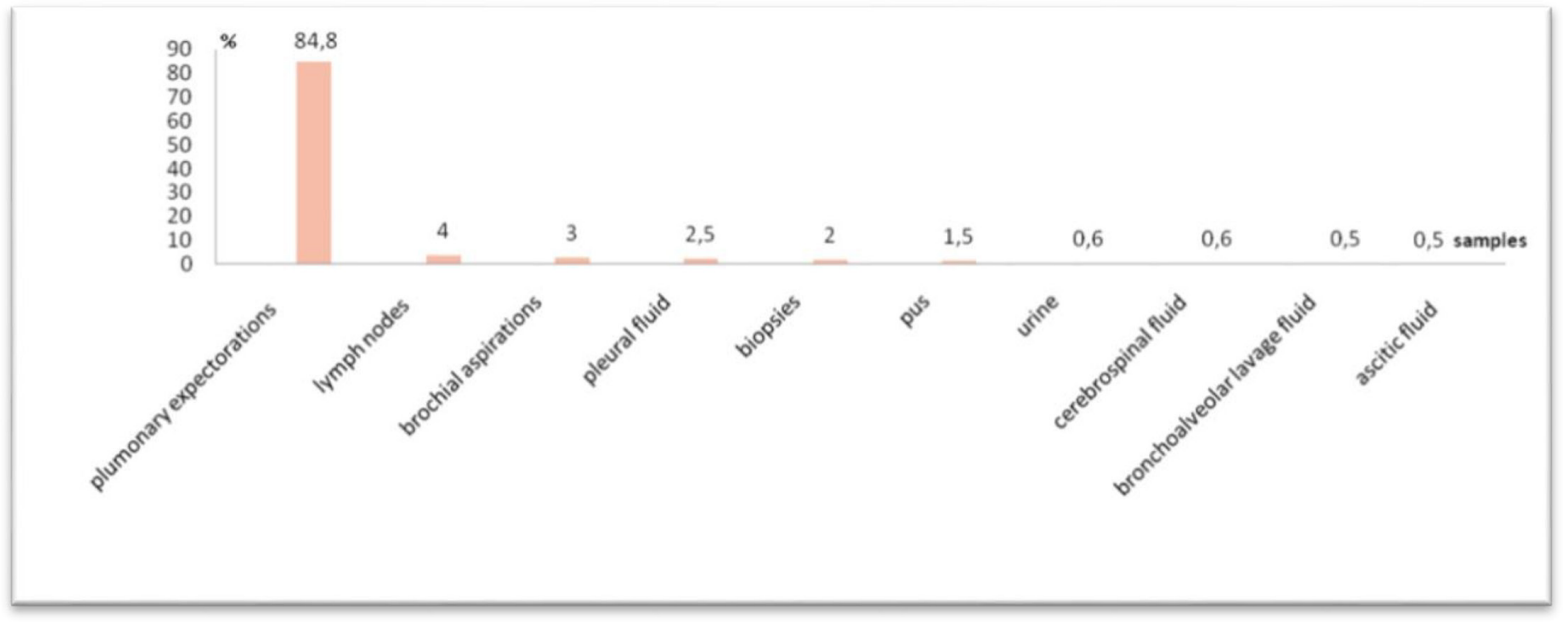
The nature of the sample collection varies depending on the location of the TB. (The nature of the samples varied according to the location of the TB: 84.8% were pulmonary expectorations, 4% were lymph nodes, 3% were bronchial aspirations, 2.5% were pleural fluid, 2% were biopsies, 1.5% were pus, 0.6% were urine, 0.6% were cerebrospinal fluid, 0.5% were bronchoalveolar lavage fluid and 0.5% were ascitic fluid).

**Fig. 2.**
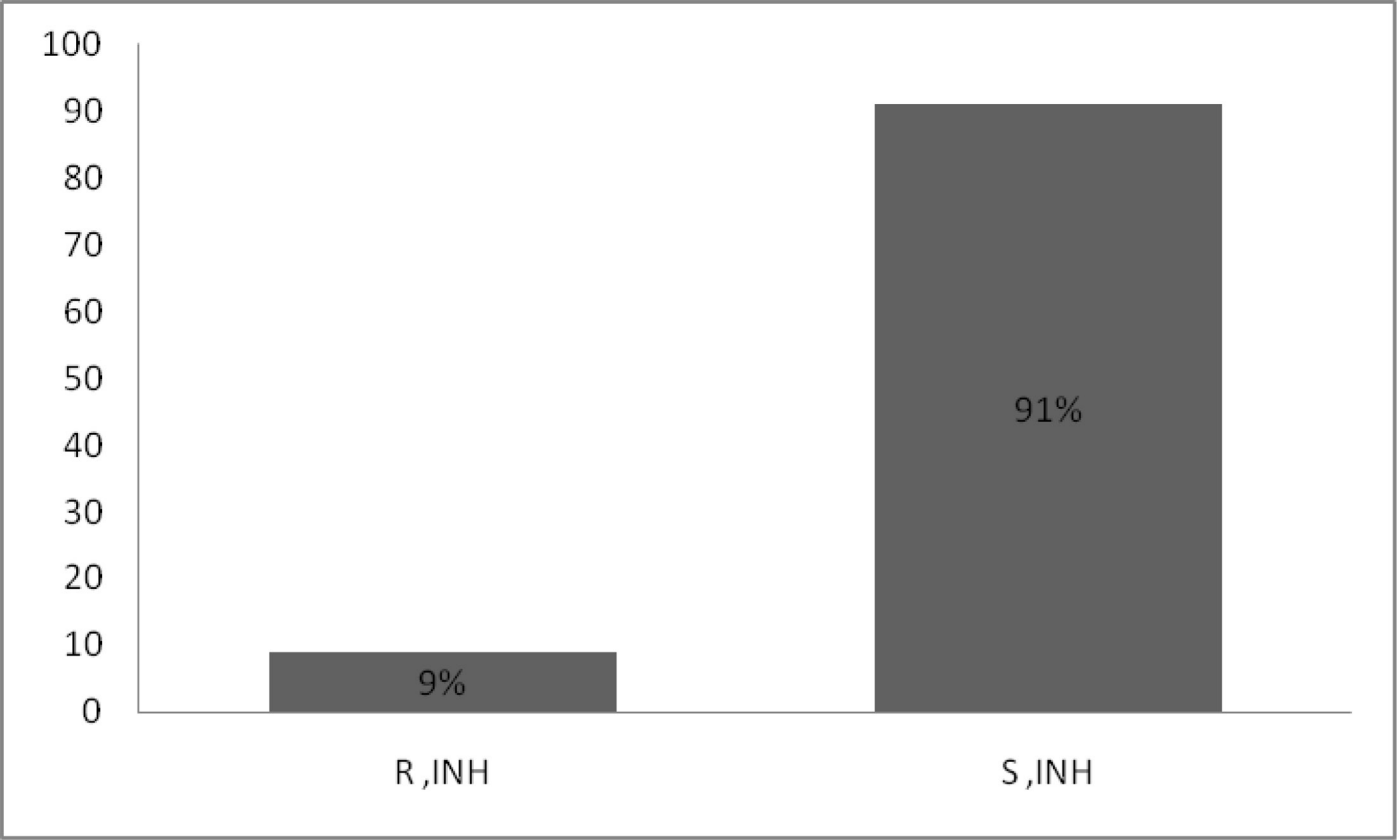
The prevalence of resistance to INH (R: INH-resistant TB; S: INH-sensitive TB). Among the 464 isolates, the prevalence of resistance to INH was 9% (43 out of 464).

A total of 464 patients diagnosed with pulmonary and extrapulmonary TB were included in the study. The mean age of the patients was 42.2 years, ranging from 8 to 88 years. Males represented 74% of the cohort, yielding a sex ratio of 2.8. Most samples (84.8%) were obtained from pulmonary sites, while extrapulmonary samples accounted for 15.2% of the total. In Fig. 1, the nature of the sample collection varies depending on the location of the TB, and the positivity rate for direct smear microscopy was 74%, whereas culture positivity reached 100%. Among the 464 isolates, INH resistance was detected in 9% (*n*=43). In Fig. 2, the prevalence of resistance to INH is shown.

**Fig. 3.**
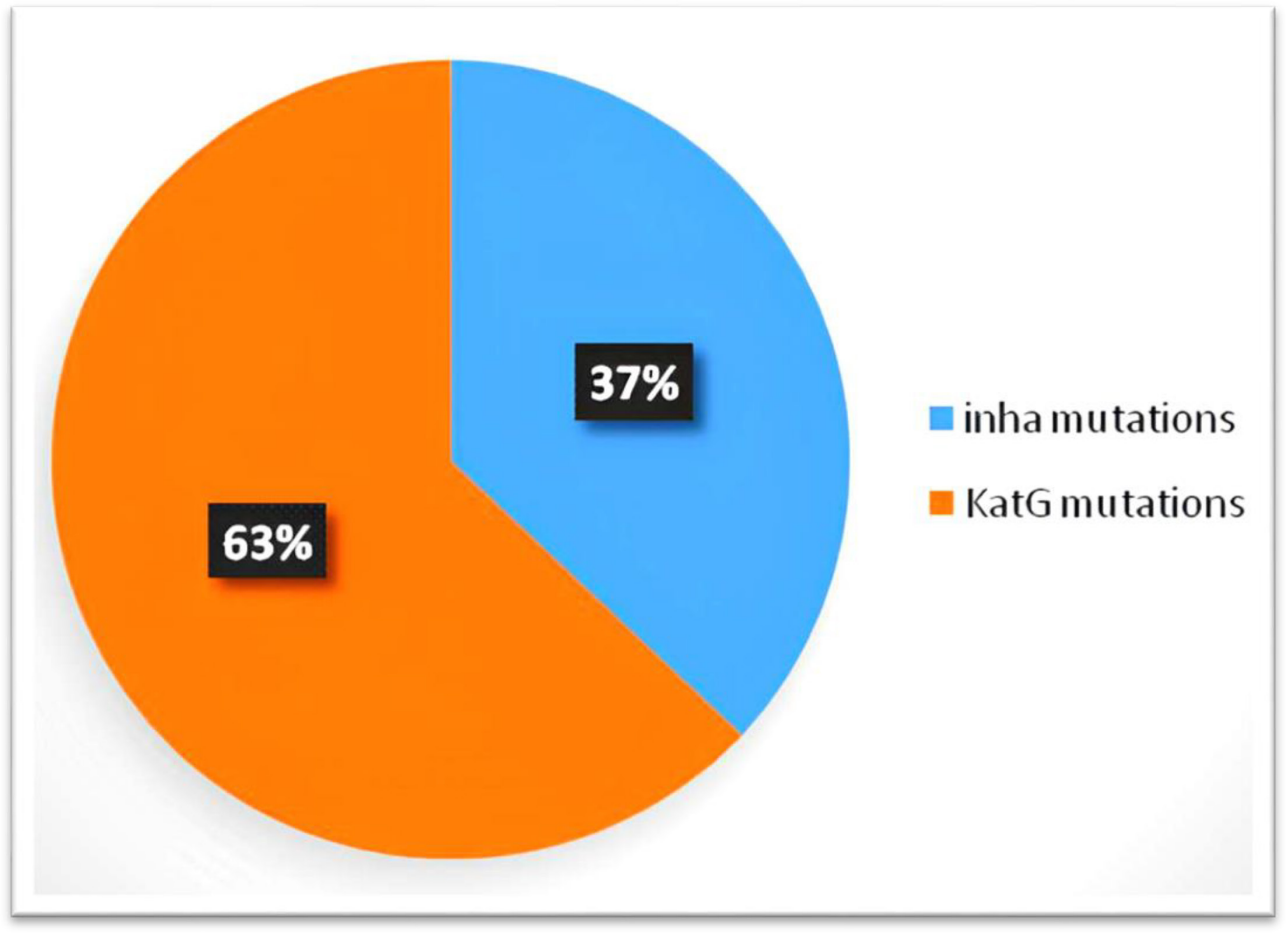
The genetic mutations observed in INH-resistant clinical isolates. (The genetic mutations observed showed that 63% of INH-resistant clinical isolates had mutations in the *katG* gene, while 37% had mutations in the *inhA* gene).

Chi-square analysis showed a statistically significant association between sex and INH resistance (*P*<0.05), with males comprising 86% of the INH-resistant group. Among those resistant to INH, genetic mutation analysis revealed that 63% (*n*=27) harboured mutations in the *katG* gene and 37% (*n*=16) in the *inhA* promoter region. In Fig. 3, the genetic mutations observed in INH-resistant clinical isolates are shown. No statistically significant association was found between the type of clinical specimen (pulmonary vs. extrapulmonary) and INH resistance (*P*>0.05). An independent samples t-test indicated no significant difference in mean age between INH-resistant and INH-sensitive patients (*P*>0.05). These findings suggest that while INH resistance is not strongly age-dependent, it is more commonly observed among male patients, and the predominance of *katG* mutations highlights the likelihood of high-level INH resistance in this population.

## Discussion

INH resistance is a growing global problem, affecting the management of TB treatment, both in drug-sensitive and MDR forms. According to the WHO, INH resistance is a global issue with variable prevalence across regions. In 2022, global data indicated a prevalence of INH resistance of 12%.

INH resistance is the most commonly encountered resistance in TB cases [7]. In our study population, the prevalence of INH-resistant TB cases is 9% (43 out of 464). This prevalence is considered high compared to that reported by Munang *et al*. in the UK (3%) [7] and Bedewi Omer *et al*. in Ethiopia (8.9%) [8] and low compared to that reported by Abdelhadi *et al*. in Chad (13%) [9], Uçar *et al*. in Turkey (12.3%) [10], Baykal *et al*. in the Van province in Turkey (12%) [11], Chevalier *et al*. in Senegal (Dakar) (18.9%) [12], Desikan *et al*. in India (27.4%) [13] and Saif Alfaresi and Hag-Ali in the United Arab Emirates (34.5%) [14]. A study conducted by the Pasteur Institute of Casablanca revealed that the prevalence of INH resistance was 12.5% among new TB cases in 2018.

According to these results, the prevalence of INH resistance in our study aligns with national and international trends, fluctuating around 9–15%.

Ramaswamy and Musser studied INH resistance in *M. tuberculosis* isolates from several geographical regions. They observed that mutations in *katG* were responsible for the majority of INH-resistant cases, accounting for ~40–60% of resistant strains, while mutations in *inhA* were less frequent, occurring in about 20–30% of cases [15].

A study by Farnia analysed INH-resistant *M. tuberculosis* isolates from several countries, including Iran, India and South Africa. They found that mutations in *katG* were the most common, but mutations in *inhA* were also present in resistant strains. The frequency of mutations in *inhA* varied by region, which has important implications for treatment strategies tailored to each local context [16].

They are responsible for ~60–70% of INH resistance cases in the country [17], whereas mutations in the *inhA* gene are less frequent compared to those in the *katG* gene in Morocco. These mutations account for about 20–30% of INH-resistant cases [18]. Based on these data and our results, these trends align with international observations, where *katG* mutations are also most frequently associated with INH resistance.

The finding of a 9% prevalence of INH resistance among *M. tuberculosis* isolates in this study is both clinically and programmatically significant. While this rate is slightly below the global average of 10–13% reported by the WHO[1], further INH surveillance of *M. tuberculosis* is recommended as treatment failure could increase if INH resistance increases over time.

The implication is that nearly one in ten patients in Morocco may be receiving ineffective first-line therapy if drug susceptibility testing is not conducted, putting them at risk of treatment failure, prolonged infectiousness and progression to MDR-TB. This is particularly concerning in high-burden settings, where delays in diagnosis or treatment modification can facilitate community-level transmission of resistant strains. The mutation profile observed 63% *katG* and 37% *inhA* and provides additional insight into resistance dynamics. The predominance of *katG* mutations, particularly the S315T substitution, is consistent with global data [19,20] and is typically associated with high-level INH resistance and loss of efficacy even at higher doses. In contrast, *inhA* mutations often confer low-level resistance and may retain some susceptibility to high-dose INH, although they also signal potential cross-resistance to ethionamide, which has therapeutic implications if second-line treatment is needed [21]. However, several limitations must be acknowledged. The study was conducted in a single centre, potentially limiting the generalizability of the results to the broader national population. Additionally, the GenoType MTBDR plus assay detects only the most common mutations in *katG* and the *inhA* promoter, which means that less frequent or novel resistance-conferring mutations may have been missed [22]. The absence of phenotypic data, such as MICs, and clinical outcome information further restricts the ability to fully assess the impact of the detected mutations on treatment efficacy.

## Conclusion

The increasing prevalence of *M. tuberculosis* complex strains resistant to one or more first-line anti-TB drugs highlights the urgent need for targeted and ongoing epidemiological surveillance. In this study, we found that INH resistance affected 9% of TB cases over the 3-year period, underscoring a significant yet under-recognized threat to TB control efforts in Morocco. Molecular analysis revealed that the majority of resistant strains carried mutations in the *katG* gene, with a smaller proportion exhibiting mutations in the *inhA* promoter region. These findings emphasize the importance of incorporating molecular diagnostics capable of detecting INH resistance even in the absence of RIF resistance into routine TB surveillance programmes. Strengthening diagnostic capacity and updating treatment protocols accordingly will be essential to curb the spread of INH-resistant TB and prevent the emergence of MDR forms.

In conclusion, while significant progress has been made in the fight against TB globally and in Morocco, drug resistance remains a major challenge that requires renewed strategies and strengthened international cooperation to eradicate the disease by 2035.

## Abbreviations

HIV: human immunodeficiency virus
INH: isoniazid
LJ: Löwenstein–Jensen
MDR: multidrug-resistant
RIF: rifampicin
TB: tuberculosis
WHO: World Health Organization
